# Intravenous Acetaminophen vs. Ketorolac in Terms of Pain Management in Prehospital Emergency Services: A Randomized Clinical Trial

**DOI:** 10.22114/ajem.v0i0.130

**Published:** 2019-05-08

**Authors:** Babak Mahshidfar, Mahdi Rezai, Saeed Abbasi, Davood Farsi, Peyman Hafezimoghadam, Mani Mofidi, Ramin Almasi, Shaqayeq Khosravi

**Affiliations:** 1. Emergency Medicine Management Research Center, Iran University of Medical Sciences, Tehran, Iran.; 2. Student Research Committee, School of Medicine, Iran University of Medical Sciences, Tehran, Iran.

**Keywords:** Acetaminophen, Emergency Medical Services, Ketorolac, Pain Management

## Abstract

**Introduction:**

Although pain management in EDs has been fully addressed in clinical trials, prehospital settings have rarely been investigated.

**Objective:**

The present study was conducted to compare the effectiveness of intravenous acetaminophen with that of ketorolac in pre-hospital pain control.

**Method:**

This randomized clinical trial (RCT) was performed at a prehospital setting during EMS missions in Tehran, Iran. The eligible candidates comprised all patients over the age of 7 years with a complaint of moderate to severe pain. The patients were randomly assigned to two groups, one receiving 30 mg of intravenous (IV) ketorolac and the other 1 g of IV acetaminophen. The pain intensity was measured using a visual analog scale (VAS) before administering the analgesic and upon admission to the ED.

**Results:**

The present study was conducted on 150 patients aged 8–81 years with a mean age of 40.4 ± 17.7, including 84 (56%) males. The mean reduction in the pain score was 14.9±8.6 in the acetaminophen group and 16.0±8.8 in the ketorolac group. Univariate analyses suggested no statistically significant differences between the two groups in terms of delta pain score (pain reduction) (P=0.429).

**Conclusion:**

Based on the obtained findings, both ketorolac and acetaminophen could be administered for pain management in prehospital settings in both traumatic and non-traumatic patients in case their contraindications are considered.

## Introduction

Emergency patients always complain about the unpleasant sensation of pain. It is believed that, the earlier the pain control is started, the more successful it appears to be. So, starting pain management by emergency medical service (EMS) technicians can help with the better control of pain in the emergency department (ED) (1, 2). Despite the possibility of using different analgesic agents in prehospital settings, as is the frequent case in some developed countries, underdeveloped and developing countries such as Iran are facing certain obstacles in this regard (3). Although pain management in EDs has been fully addressed in clinical trials, prehospital settings have rarely been investigated. This type of studies can therefore provide documented information required for determining appropriate pain management methods at the pre-hospital emergency stage. The present study was therefore conducted to compare the effectiveness of intravenous acetaminophen with that of ketorolac in pre-hospital pain control.

## Methods

### Study design

This randomized clinical trial (RCT) was performed at a prehospital setting during EMS missions in Tehran, Iran. After approving the study protocol by ethics committee of Iran University of Medical Sciences, sampling was performed from September 2011 until March 2012. After signing informed consent forms, the patients were included in the study. The Declaration of Helsinki was also followed throughout the study by the researchers.

### Study population

The eligible candidates comprised all patients over the age of 7 years with a complaint of moderate to severe pain and able to respond to the research questions. The exclusion criteria consisted of altered levels of consciousness, pain with cardiac origins, gastrointestinal bleeding and a history of allergy to acetaminophen or ketorolac.

### Intervention and data collection

A checklist was prepared in two parts; the first part included variables associated with demographic characteristics of the patients, i.e. age and gender, and the second involved pain characteristics, i.e. the pain cause and intensity before and after the intervention. The patients were randomly assigned to two groups, one receiving 30 mg of intravenous (IV) ketorolac and the other 1 g of IV acetaminophen. The pain intensity was measured using a visual analog scale (VAS) before administering the analgesic and upon admission to the ED.

### Definition

A pain reduction of over 13 mm was considered acceptable and a reduction of over 30 mm clinically significant. A VAS pain intensity of 0–35 was considered mild, 36–65 moderate and 66–100 severe (4).

### Statistical analysis

To analyze the data, the Chi-square test was used to compare the categorical variables, and the independent t-test and Pearson correlation to compare the numerical variables. ANCOVA was also used for the association of delta pain reduction with type of drug and another covariate. Moreover, the Shapiro–Wilk test was used to test the normality assumption. P<0.05 was set as the level of statistical significance. The statistical analysis was performed in SPSS.

## Results

The present study was conducted on 150 patients aged 8–81 years with a mean age of 40.4 ± 17.7, including 84 (56%) males. Table 1 summarizes the baseline characteristics of the study patients. The mean reduction in the pain score was 14.9±8.7 in the acetaminophen group and 16.0±8.8 in the ketorolac group. Univariate analyses suggested no statistically significant differences between the two groups in terms of delta pain score (pain reduction) (P=0.429). Table 2 presents the pain score of the study patients at different time points.

**Table 1:** Baseline characteristics of the study patients

**Variable**	**Acetaminophen (n=75)**	**Ketorolac (n=75)**	**P**
**Mean age (year)**	40.5±17.4	40.3±18.1	0.938
**Gender**			0.869
Male	41±54.7	43±57.3	
Female	34±45.3	32±42.7	
**Cause of pain**			0.623
Trauma	32±42.7	36±48.0	
Others	43±57.3	39±52.0	
**Pain intensity**			0.106
Moderate	48±64.0	58±77.3	
Severe	27±36.0	17±22.7	

**Table 2:** The VAS pain score of the study patients at different time points

**Time points**	**Acetaminophen (n=75)**		**Ketorolac (n=75)**		**p**
	**Mean (SD)**	**Median (IQR)**	**Mean (SD)**	**Median (IQR)**	
**Before analgesic administration**	61.8 (11.7)	60.0 (16.0)	57.8 (10.5)	58.0 (14.0)	0.031
**Upon admission to the ED**	46.9 (15.1)	46.0 (20.0)	41.8 (13.0)	40.0 (16.0)	0.028
**Time interval between prescribing and pain assessment in EMS (min)**	23.8 (5.4)	23.0 (6.0)	23.5 (3.7)	23.0 (5.0)	0.752
**Pain reduction per minute**	0.63 (0.37)	0.67 (0.32)	0.69 (0.38)	0.65 (0.33)	0.302

**Table 3:** The mean score (SD) of the delta pain and the pain reduction rate in the study patients based on the baseline characteristics

	**Δ Pain score**		**P**	Pain reduction per minute		**P**
	**Acetaminophen (n=75)**	**Ketorolac (n=75)**		**Acetaminophen (n=75)**	**Ketorolac (n=75)**	
**Gender**						
Male	13.4 (8.0)	14.9 (8.0)	0.395	0.58 (0.37)	0.65 (0.35)	0.389
Female	16.6 (9.2)	17.5 (9.8)	0.716	0.68 (0.36)	0.74 (0.43)	0.515
**P**	0.105	0.271		0.207	0.313	
**Pain cause**						
Trauma	17.4 (8.2)	18.1 (5.6)	0.677	0.75 (0.32)	0.78 (0.28)	0.587
Non-trauma	13.0 (8.6)	14.1 (10.8)	0.617	0.54 (0.39)	0.60 (0.45)	0.483
**P**	0.027^*^	0.014^*^		0.042^*^	0.037^*^	
**Pain intensity**						
Moderate	16.1 (7.6)	15.1 (6.5)	0.472	0.70 (0.33)	0.66 (0.31)	0.435
Severe	12.7 (10.0)	19.2 (14.0)	0.083	0.48 (0.41)	0.80 (0.58)	0.040^*^
**P**	0.111	0.012^*^		0.256	0.340	

* Statistically significant

Table 3 presents the delta pain score and the rate of pain reduction in the study patients based on the baseline characteristics. The results suggest that both medicines could significantly relieve traumatic pains compared to non-traumatic pains (P<0.05).

Moreover, ketorolac was found to be more effective in reducing the intensity of severe pains than moderate pains. Ketorolac was also found to be more effective in reducing the pain score (P=0.083) and the rate of pain reduction (P=0.040) in case of severe pains compared to acetaminophen (Table 3).

Delta pain reduction was found to be more significantly correlated with age in the ketorolac group (r=0.262, P=0.023) compared to in the acetaminophen group (r=0.141, P=0.229).

ANCOVA suggested that pain intensity (F=9.2, P=0.003) and gender (F=6.1, P=0.015) were statistically significant and age (F=2.9, P=0.091) marginally significant for delta pain score as the dependent variable, although the type of medicine was insignificant (F=0.52, P=0.471).

## Discussion

The present research found both ketorolac and acetaminophen to clinically relieve pain in pre-hospital settings, and to be more effective in relieving traumatic pains than non-traumatic pains. In severe pains, ketorolac was found to cause a higher reduction in the pain score compared to acetaminophen. Conversely, acetaminophen was more effective in managing moderate pains than sever pains. These medicines were found to be more effective in pain reduction in younger ages, and this finding was more significant in the ketorolac group compared to in the acetaminophen group. These medicines were also more effective in men than in women.

A systematic review on prehospital pain management of trauma patients by Dijkstra et al. found paracetamol to be an effective analgesic both orally and intravenously, which is consistent with the present findings (5). The efficacy of acetaminophen in pain management has been addressed in several RCTs, most of which reported a paracetamol-associated pain reduction, although it was not clinically effective (6–9). The present study appears to have pioneered the IV administration of paracetamol for pain management, which caused a 13-mm VAS pain reduction. Although acetaminophen has not been yet conclusively compared with opioids in terms of pain relief in pre-hospital settings, they have been reported to be equally effective in some studies (9, 10).

In contrast to the obtained results for the effectiveness and safety of acetaminophen, Dijkstra et al. and Macintyre et al. found that non-steroidal anti-inflammatory drugs to cause mixed effects in terms of effectiveness, as they cause many contraindications and serious side effects. They did not therefore recommend these drugs for prehospital settings given the restricted possibilities for adequate risk assessment on potential contraindications and side effects (5, 11); nevertheless, ketorolac showed proper effects on prehospital traumatic pain management in the present study. It is worth noting that the study patients were not followed up for possible side-effects during and after hospitalization, and those with known contraindications were excluded. The results obtained in this regard by Hoogewijs et al., Whitefield et al. and Woo et al. appear in line with the present results (6, 7, 12). Hoogewijs et al. reported that IV diclofenac and IV propacetamol caused an equal statistically-significant pain reduction of below 2 points based on the VAS, suggesting that both medicines were ineffective (6). It is consistent with the present research attributing a pain relief of more than 30 mm (3 points) to ketorolac, although the 13 mm decrease based on the VAS could be considered acceptable. The data collected are recommended to be interpreted by considering more findings such as patient satisfaction.

### Limitation

Given pain measurement as a subjective criterion in subjects, factors such as a desire for more pain relief by using higher doses of painkillers, the patient’s failure to comprehend the questionnaire’s items and a childish fear of reinjections can influence the reliability of these criteria, the actual pain reduction and its associated outcomes. Given that possible side-effects during and after hospitalization were not investigated in the present study, the findings are recommended to be interpreted and analyzed cautiously.

## Conclusions

Based on the obtained findings, both ketorolac and acetaminophen could be administered for pain management in prehospital settings in both traumatic and non-traumatic patients in case their contraindications are considered. The effectiveness of both drugs also appear to decrease with age.
